# The Stress Detection and Segmentation Strategy in Tea Plant at Canopy Level

**DOI:** 10.3389/fpls.2022.949054

**Published:** 2022-07-06

**Authors:** Xiaohu Zhao, Jingcheng Zhang, Ailun Tang, Yifan Yu, Lijie Yan, Dongmei Chen, Lin Yuan

**Affiliations:** ^1^College of Artificial Intelligence, Hangzhou Dianzi University, Hangzhou, China; ^2^School of Information Engineering, Zhejiang University of Water Resources and Electric Power, Hangzhou, China

**Keywords:** tea green leafhopper, anthracnose, sunburn, deep learning, image processing

## Abstract

As compared with the traditional visual discrimination methods, deep learning and image processing methods have the ability to detect plants efficiently and non-invasively. This is of great significance in the diagnosis and breeding of plant disease resistance phenotypes. Currently, the studies on plant diseases and pest stresses mainly focus on a leaf scale. There are only a few works regarding the stress detection at a complex canopy scale. In this work, three tea plant stresses with similar symptoms that cause a severe threat to the yield and quality of tea gardens, including the tea green leafhopper [*Empoasca (Matsumurasca) onukii* Matsuda], anthracnose (*Gloeosporium theae-sinensis* Miyake), and sunburn (disease-like stress), are evaluated. In this work, a stress detection and segmentation method by fusing deep learning and image processing techniques at a canopy scale is proposed. First, a specified Faster RCNN algorithm is proposed for stress detection of tea plants at a canopy scale. After obtaining the stress detection boxes, a new feature, i.e., RGReLU, is proposed for the segmentation of tea plant stress scabs. Finally, the detection model at the canopy scale is transferred to a field scale by using unmanned aerial vehicle (UAV) images. The results show that the proposed method effectively achieves canopy-scale stress adaptive segmentation and outputs the scab type and corresponding damage ratio. The mean average precision (mAP) of the object detection reaches 76.07%, and the overall accuracy of the scab segmentation reaches 88.85%. In addition, the results also show that the proposed method has a strong generalization ability, and the model can be migrated and deployed to UAV scenarios. By fusing deep learning and image processing technology, the fine and quantitative results of canopy-scale stress monitoring can provide support for a wide range of scouting of tea garden.

## Introduction

Tea is an important economic crop whose demand is increasing worldwide (Xia et al., 2017; Bora et al., 2019; Fang et al., 2019). However, the tea production is affected by diseases and pest infestations, which poses a severe threat to the yield and quality of tea leaves. Currently, there are nearly 1,000 types of tea plant pests and more than 380 tea plant diseases that have been reported globally. These diseases and pests lead to yield losses as high as 43% (Gnanamangai and Ponmurugan, 2012; Sun et al., 2019; Roy et al., 2020). An effective detection and identification of major tea plant diseases and pests can provide a guide map for prevention and control. In addition, it can also assist in reducing the environmental pollution caused by the excessive application of pesticides (Sankaran et al., 2010; Hu et al., 2019a). It is noteworthy that the detection of tea plant diseases and pest infestations is also important for high-throughput phenotypic analysis during tea plant breeding. The conventional phenotyping based on manual inspection is prone to subjective errors. Currently, there is a lack of efficient methods for detecting disease-resistant tea plant phenotypes. Therefore, non-destructive high-throughput methods for detecting tea pests and diseases are highly desired (Mahlein et al., 2019).

Due to the rapid popularization of portable cameras, the researchers are able to easily acquire the images of plant diseases and pests. There are various works presented in literature that combine the image processing techniques with machine learning methods for the detection of pests and diseases. Patil and Zambre (2014) extracted the texture, shape, color, and other features, and then used a support vector machine (SVM) for classifying various cotton leaf diseases and pests with an accuracy of 96.66%. Waghmare et al. (2016) used texture features and SVM to classify the grape leaf downy mildew and black rot with an accuracy of 96.6%. Ramesh et al. (2018) used Hu moments, Haralick texture, and color features for classifying papaya leaves based on random forest (RF). The classification accuracy of this method is approximately 70%. Shrivastava and Pradhan (2021) only used color features and SVM to classify the rice diseases, including bacterial blight, rice blast, sheath blight, and healthy leaves, with a classification accuracy of 94.65%. Xian and Ngadiran (2021) used features, such as Haralick texture, hue saturation value (HSV) histogram, and color moments to classify tomato leaves with an extreme learning machine. The classification accuracy of this method reaches 84.94%, which is better as compared to the method that uses the RF algorithm. The aforementioned methods for classifying plant pests and diseases based on machine learning are usually suitable for simple scenarios, such as single plant and single species of pests and diseases. However, these methods are unable to perform efficiently in complex real-world scenarios. In addition, the machine learning models are highly dependent on the training samples.

As compared with machine learning, the deep learning methods automatically realize feature learning based on given data. This enables the researchers to build end-to-end models for plant disease and pest detection (Noon et al., 2020). Tian et al. (2020) compared the results of deep learning and machine learning in the classification of citrus pests and diseases. The results show that the classification accuracy of convolutional neural network (CNN; 95.83%) was significantly higher than SVM (87.65%). Due to the strong generalization of deep learning, many researchers have realized the classification of plant leaf diseases and pests based on CNN, including, but not limited to tea, wheat, rice, ginkgo, walnut, coffee, cucumber, tomato, apple, and banana leaves, achieved high classification accuracy (Hu et al., 2019b; Anagnostis et al., 2020; Esgario et al., 2020; Karthik et al., 2020; Li et al., 2020; Zhong and Zhao, 2020). In addition to the application of deep learning in the classification of plant leaf diseases and pests, various works have been presented for locating and assessing the damaged areas. Liu and Wang (2020) optimized YOLOv3 based on feature fusion and other methods for detecting tomato leaf diseases and pests with a mean average precision (mAP) of 92.39%. Fuentes et al. (2017) used a faster region-based convolutional neural network (Faster RCNN), region-based fully convolutional network, and single-shot multi-box detector for detecting the diseases and pests on tomato leaves. The results show that Faster RCNN performs better in multi-stress types. The transfer learning effectively shortens the training time and achieves suitable results without requiring large-scale datasets and has been widely used in plant disease detection (Selvaraj et al., 2019). Currently, the research on plant diseases and pests mainly focuses on a leaf scale. There are very few works that consider the disease and pest detection at the canopy level. The leaf level detection of diseases and pests always requires leaf sampling, or taking tight shots of leaves, which limited its application in field scouting scenarios, such as using the near-ground unmanned aerial vehicle (UAV) system as demonstrated in our study. And the canopy level detection is able to provide the distribution information of disease/pest occurrence and incidence, which is important to guide the prevention practices, especially for smart sprayers. The key point of detection at the canopy scale is to realize the automatic identification of the range of diseases and pest lesions, and the estimation of damage ratio, which is conducive to determining the type of stress and the degree of incidence, so as to carry out precise prevention and control.

In the tea planting areas that are mainly used for green tea production, tea green leafhopper (GL) and anthracnose (AH) are the most frequent leaf disease and pest during the period of May–June. During the same period, the tea gardens are also susceptible to the damage of leaf sunburn (BR). This study focuses on the detection of three aforementioned tea plant stresses that can occur simultaneously in the tea garden that have similar symptoms. The core motivation is to propose an intelligent detection method of tea plant stresses on canopy level that is able to facilitate non-destructive high-throughput field scouting. The major contributions of this work are as follows:

1. An image acquisition of tea plant diseases and pests including GL, AH, and BR is performed to form a dataset for multi-scale recognition.
2. A canopy-level scab detection and segmentation strategy that synergizes deep learning and image processing is proposed.
3. A specified Faster RCNN algorithm is proposed and compared with YOLO v3 for tea stresses detection.
4. A new feature, the RGReLU, is proposed and used in stresses segmentation, which thus constituted a light-weighting strategy, and compared with other features based on RGB and HSI color space.
5. The feasibility of transferring the established canopy level model to the UAV images is verified, which indicated a promising capability in automatic scouting and detecting of diseases, pests, and other stresses in tea gardens.

## Experiments and Methods

### Data Collection

**Figure 1:**
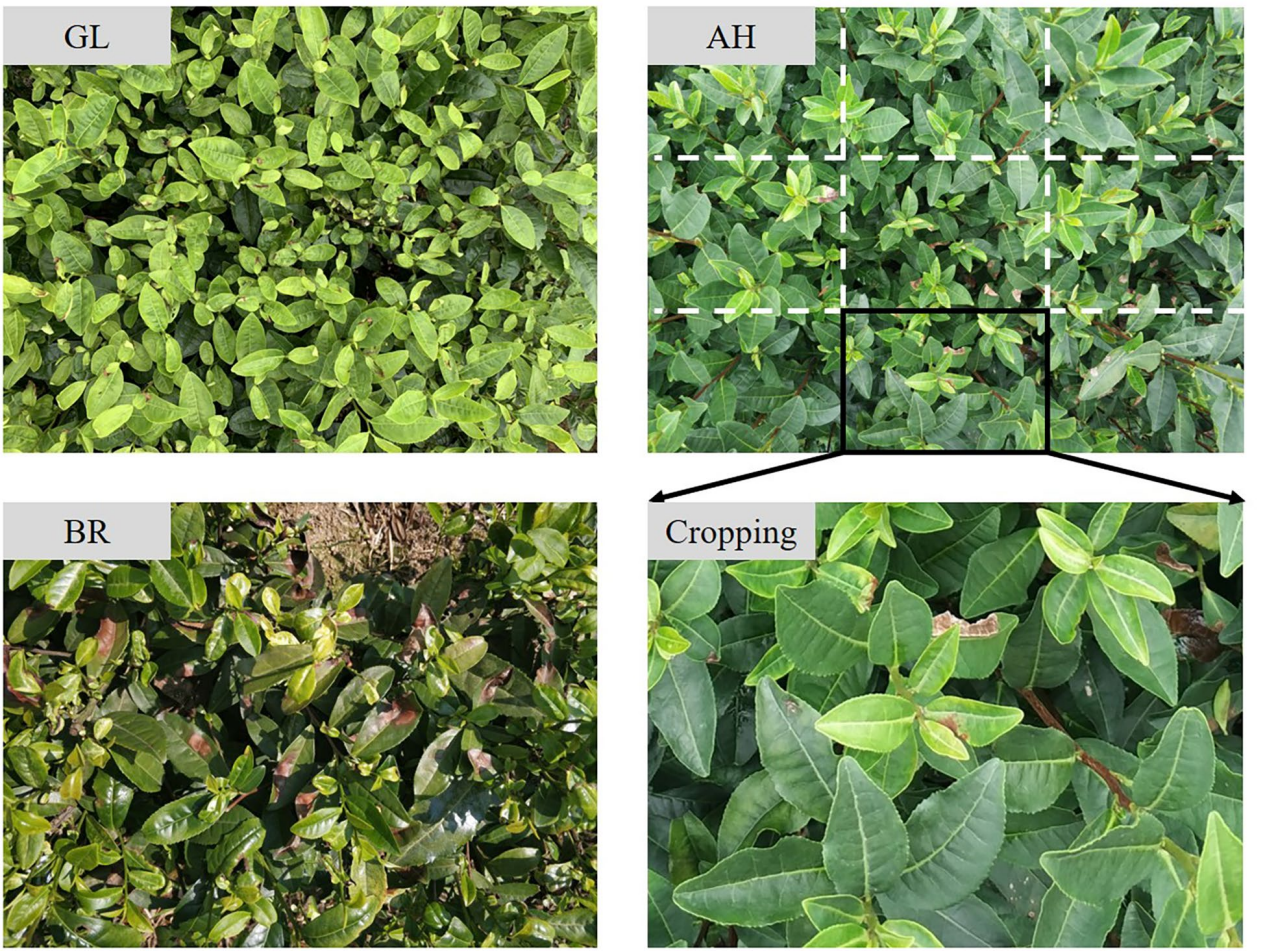
The RGB image of tea plant stresses.

The tea leaf samples of GL, AH, and BR are collected from the experimental tea gardens of the Chinese Academy of Agricultural Sciences, Hangzhou, Zhejiang, China. The symptom of these three types of stresses is similar, i.e., irregularly shaped reddish-brown areas. However, as the control strategies are quite different, the confusion among them may lead to serious consequences. In this work, the RGB images of tea plant stresses are captured using MI 6 smartphone camera (Sony, with resolution of 4,032 × 3,016) and iPhone XR smartphone camera (Sony, with a resolution of 4,032 × 3,024) in the fields. Since the tea plant stress area is too small in the image, this study firstly crops the original image during preprocessing, as presented in Figure 1. The number of stressed RGB images obtained in this work are 122 (AH), 151 (GL), and 198 (BR). After cropping, 2,375 images containing lesions are screened and retained, of which 681 are AH, 1042 are GL, and 652 are BR, as presented in Table 1. The dataset, i.e., TEAIMAGE, is divided into training, validation, and test sets according to the ratio of 7:2:1.

**Table 1:** The RGB image information regarding tea plant stresses.

Stress type	Number of original images	Number of images containing stress scabs	Camera model
AH	122	681	MI 6
GL	151	1,042	iPhone XR
BR	198	652	MI 6

### Construction of a Stepwise Segmentation Method for Tea Plant Stress at the Canopy Scale

**Figure 2:**
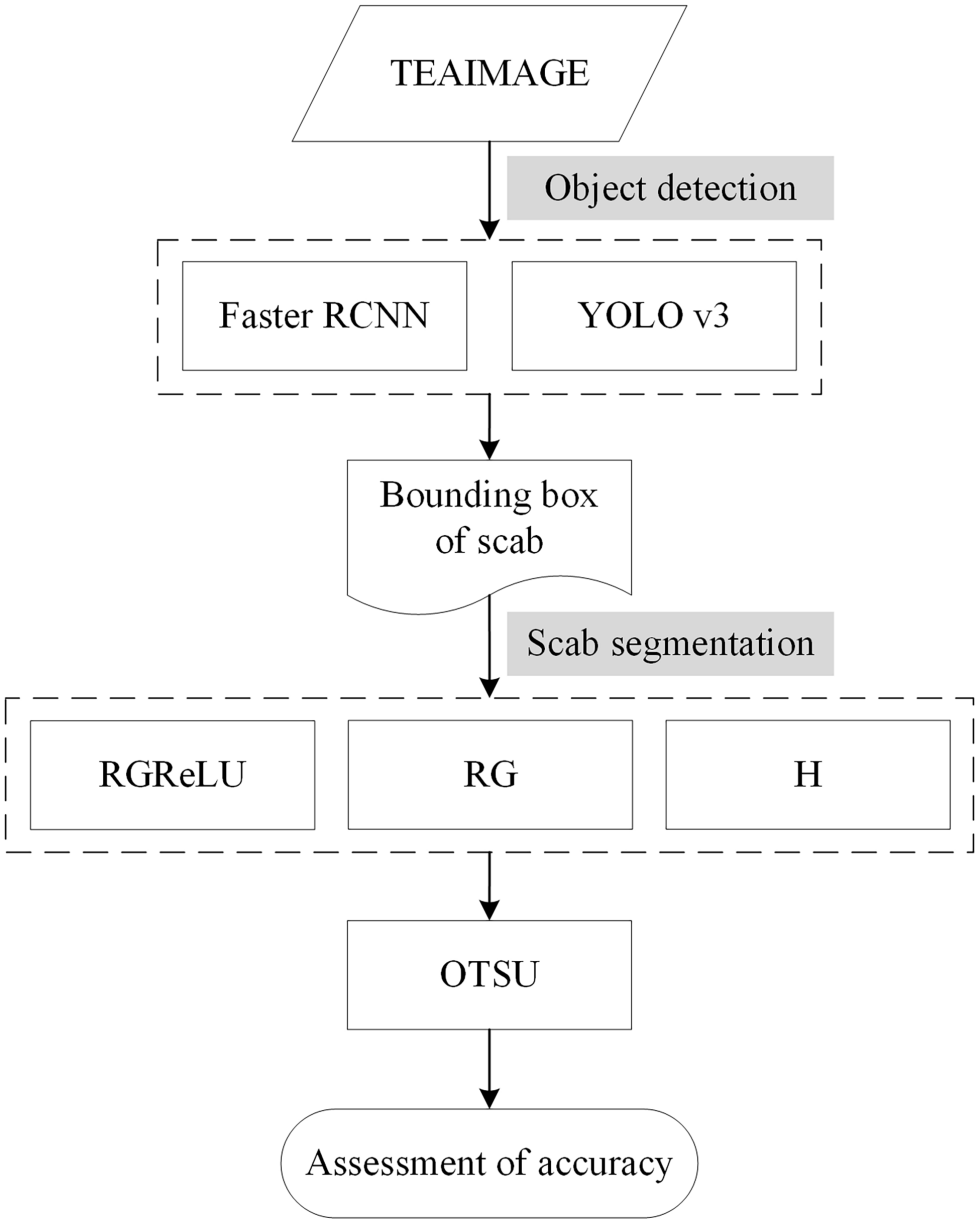
The workflow of the proposed method for detecting tea plant stresses at canopy level.

In this work, a stepwise segmentation method of tea plant stresses is proposed by combining the object detection algorithm based on deep learning and image segmentation algorithm, as presented in Figure 2. In complicated real-world scenarios, the shape and size of the scabs are different among images. First, the object detection algorithm estimates the location of the scab. Then, the image processing technique is used to achieve fine segmentation of the scab area. This stepwise strategy effectively reduces the complexity of tea plant stress segmentation in practical scenarios. According to this strategy, the scab position in the image is first located, and the stress type is determined. Second, the scab images from the detected regions are extracted and used as the input data of subsequent lesion segmentation module. Finally, the image is binarized to achieve fine scab segmentation using Otsu’s method. This stepwise strategy enables automatic detection and severity assessment of tea plant diseases, pests, and other stresses at the canopy level.

### Object Detection Algorithm Based on Deep Learning

In the object detection step, the Faster RCNN algorithm was specified according to the traits of the tea plant canopy, and compared with the classical single-stage target detection algorithm, i.e., YOLO v3.

#### Specified Faster RCNN

**Figure 3:**
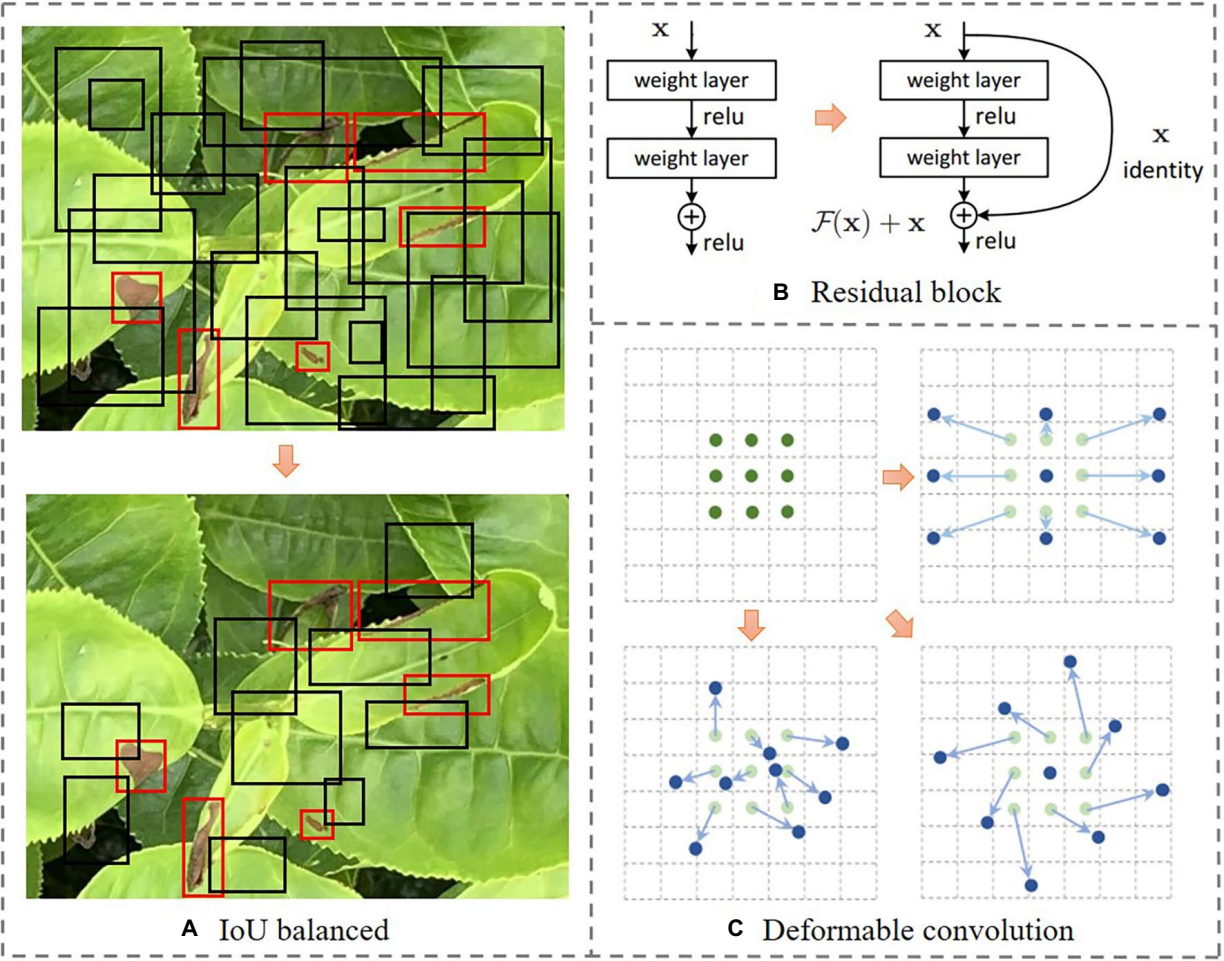
The demonstration of ideas in the specification of faster region-based convolutional neural network (Faster RCNN) in stress detection of tea plant canopy. The description of parts **A–C** is the title of its corresponding sub drawing.

The region-based convolutional neural network (RCNN) uses the selective search method to generate several candidate regions in an image. A CNN is then used to extract the features from each candidate region. Finally, these features are used as the input of SVM and linear regression model for category determination and position refinement, respectively. The Fast RCNN is a more sophisticated form of RCNN, which uses a multi-task loss function for performing classification and regression tasks based on CNN. As a simplified framework for target detection, the Faster RCNN adopts the region proposal network (RPN) instead of the selective search method. In this framework, a proposal window is generated in the convolutional feature layer of RPN by setting anchor boxes at different scales for achieving an end-to-end object detection (Ren et al., 2017). In this work, in order to improve the performance in terms of stress detection of tea plant canopy, the Faster RCNN is specified in three perspectives. First, intersection over union (IoU)-balanced sampling is added in the RPN stage. Second, ResNet101 is used as the backbone network. Third, the convolution kernels are replaced with the deformable convolution kernels, as presented in Figure 3.

##### Specification 1: Adopting IoU-Balanced Sampling in RPN Stage

**Figure 4:**
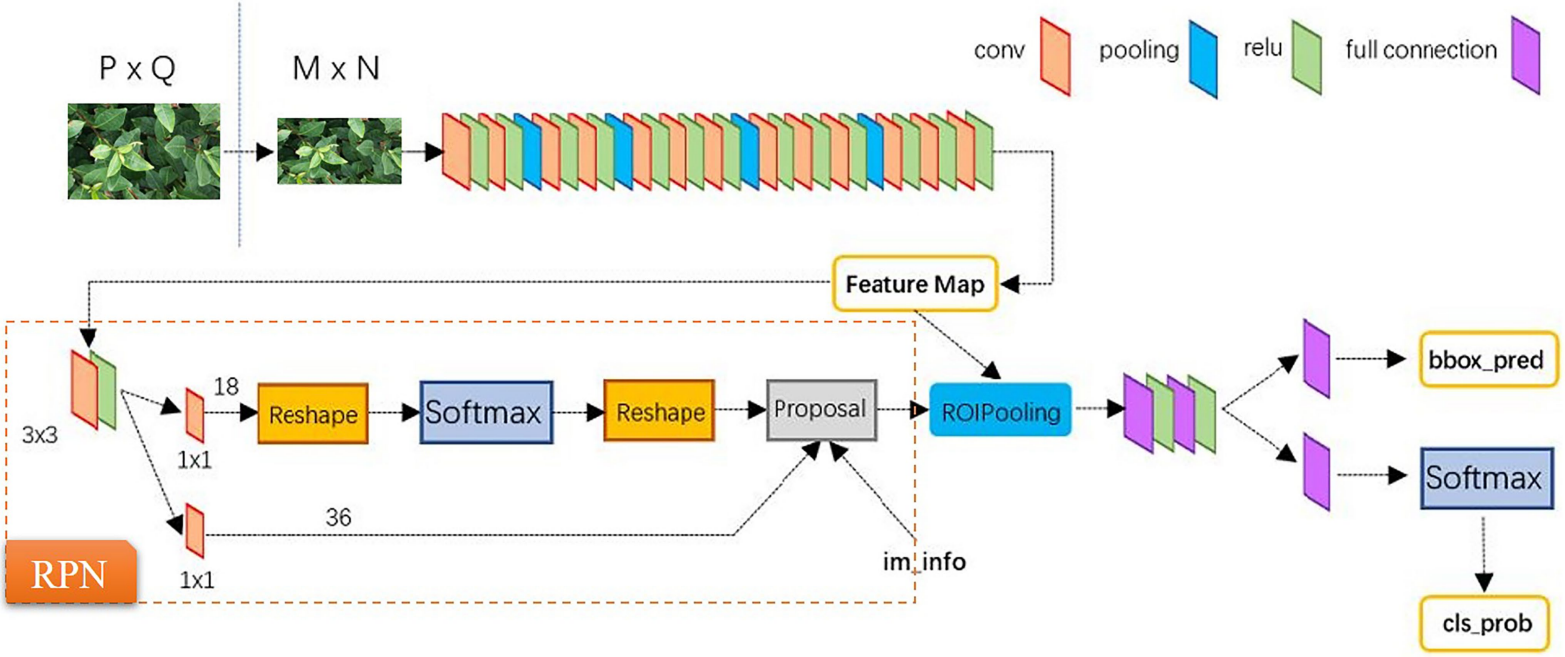
The structure of Faster RCNN.

As an important component of Faster RCNN, RPN implements the shared convolution features. This greatly improves the generation speed and localization accuracy of the detection boxes. The RPN in Faster RCNN is divided into two branches. The first branch classifies the anchor box to determine the positive or negative samples. The second branch calculates the offset of the anchor box, as presented in Figure 4. Please note that the proposal layer combines the offset of the positive anchor box and the corresponding bounding box to generate a proposal box and filters is based on IoU. As the stress area usually only occupies a small portion of an image, while the background occupies a large proportion, the random sampling may select a large number of easy samples and small number of hard samples during the generation of candidate boxes. The selection probability for each sample under random sampling is expressed as follows:


(1) $$p=\frac{N}{M}$$

where, *p* denotes the selection probability, *N* denotes the number of negative samples, and *M* denotes the number of corresponding candidates. In order to increase the selection probability of hard negatives, this work adopts the IoU-balanced sampling method proposed by Pang et al. (2019). This method starts by splitting the sampling interval into *K* bins based on IoU. It requires that the *N* negative samples are equally distributed in each bin. This guarantees a uniform selection of samples. The selection probability under the proposed IoU-balanced sampling is mathematically expressed as follows:


(2) $$p_{k}=\frac{N}{K}\times \frac{1}{M_{k}},k\in \left(0,K\right)\;$$

where, *M_k_* denotes the number of sampling candidates in the interval denoted by *k*. The experimental results show that the model performance is not sensitive to *k* (Pang et al., 2019). The parameter *k* is set to 3 based on the preliminary tests.

##### Specification 2: Selection of ResNet101 as the Backbone Network

The selection of backbone network is crucial for the performance of a model. In this work, ResNet101 is used as the backbone network of the Faster RCNN based on a preliminary analysis. Please note that the traditional backbone network degenerates as the number of layers in a network increases, i.e., with an increase in the depth of the network, the accuracy of the model saturates, and then begins to decline. The ResNet uses direct connections for connecting different layers of a network. This enables the network to overcome the loss of information caused during the forward propagation and ensures that a deeper network extracts more feature information as compared to a shallow network and avoids the gradient dispersion and network degradation caused due to the depth of the network (He et al., 2016). As presented in Figure 3B, the residual block directly adds the output before the previous layer and the output of the current layer. This result is then used as the input of the activation function, and is expressed as:


(3) $$y=F\left(x,\left\{W_{i}\right\}\right)+x$$

where, *x* and *y* denote the input and output vectors, $F=W_{2}\sigma \left(W_{1}x\right)$ in which σ denotes the rectified linear unit (ReLU). The identity shortcuts in the residual blocks realize the combination of features at different resolutions and integrate the low-level semantics of a shallow layer and the high-level semantics of a deep layer to strengthen the model performance. On the other hand, the identity shortcuts allow the model to independently perform a non-linear transformation or transfer upper-layer information during the training process, or combine the two for building a more flexible network (Lin et al., 2017).

##### Specification 3: Replacement of Convolution Kernels With the Deformable Convolution Kernels

During the detection of tea plant stresses, the dynamic shape and size of the scabs often lead to poor stress detection. The traditional feature operators and data enhancement methods only assist the model to adapt the existing known geometric transformations but are barely used in the unknown scenarios. In order to effectively address this problem, this work uses the deformable convolution kernel to replace the original convolution kernel of ResNet, thereby allowing the sampling points to diffuse into irregular shapes, as presented in Figure 3C. The deformable convolution is mathematically expressed as follows (Zhu et al., 2019):


(4) $$y\left(p\right)=\overset{K}{\underset{k=1}{\sum }}w_{k}\cdot x\left(p+p_{k}+\Delta p_{k}\right)\cdot \Delta m_{k}\;$$

where, $\Delta p_{k}$ and $\Delta m_{k}$ denote the learnable offset and modulation scalar for the *k*-th location, respectively. The *w_k_* and *p_k_* denote the weight and pre-specified offset for the *k*-th location. The *x(p)* and *y(p)* denote the features at location *p* from the input feature maps *x* and output feature maps *y,* respectively.

#### YOLO v3 Algorithm for Comparison

In addition to Faster RCNN, YOLO v3 algorithm is also used for performing detections. Contrary to the above two-staged object detection algorithm based on candidate regions, YOLO is a single-stage target detection algorithm that does not require the candidate regions. The core idea is to divide an image into an *N* × *N* grid. Each grid is responsible for detecting and localizing all the target objects existing inside it. In YOLO v3, DarkNet53 is used as the backbone network, which mines deep details in the image. Moreover, the former uses logistic regression instead of SoftMax classifier to achieve multi-label classification. Using the feature pyramid structure as a reference, YOLO v3 enlarges the size of the high-level feature maps and integrates it with the low-level feature maps. The new feature maps not only contain rich semantic information, but also have more pixels. Consequently, for small and dense targets, the detection effect is significantly improved, and the fast detection speed is achieved at the same time (Redmon and Farhadi, 2018).

### Stress Segmentation Method Based on Image Processing Technology

**Figure 5:**
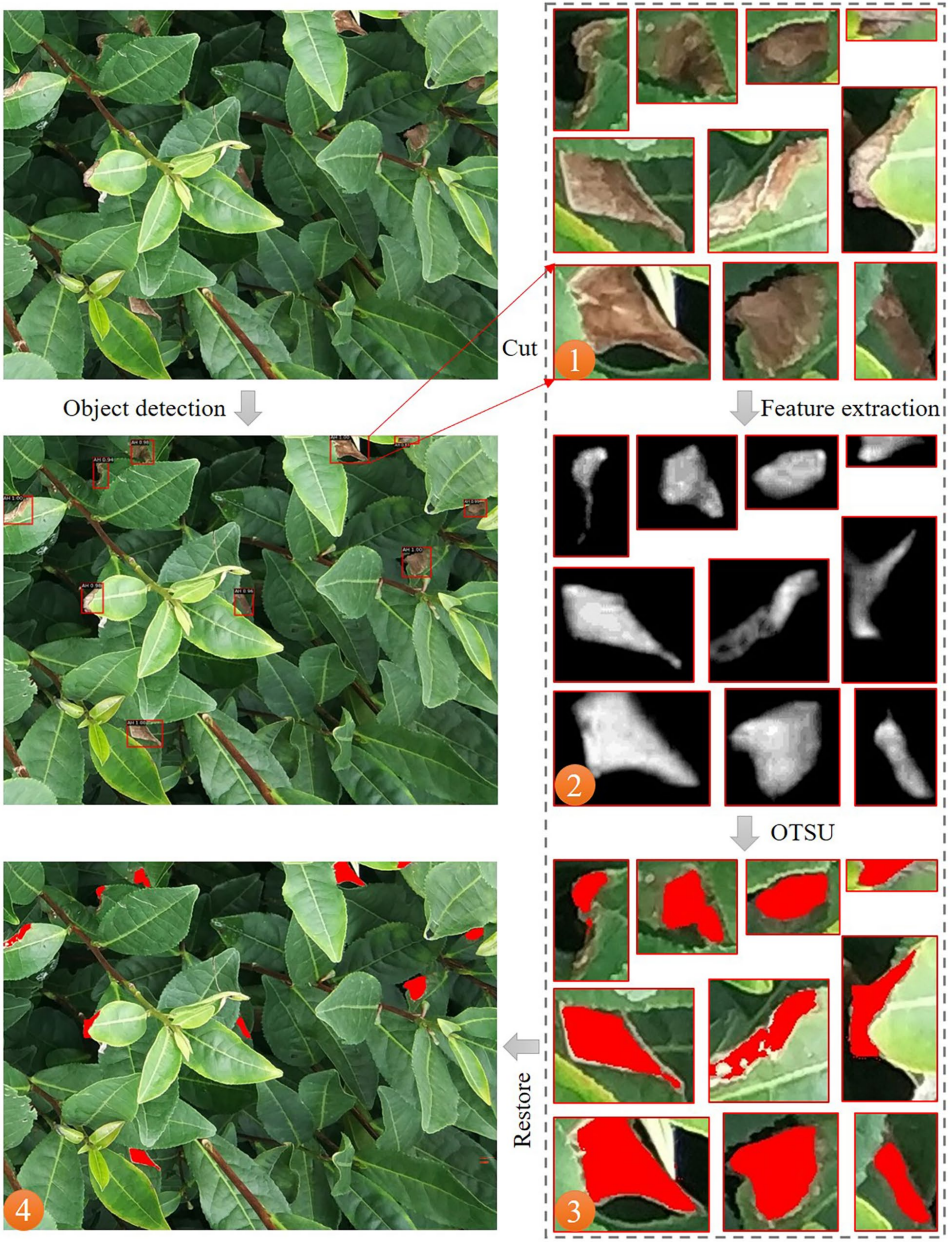
The schematic diagram of the tea plant stress segmentation method at the canopy scale.

In this part, the scab area will be segmented based on the results of the above object detection algorithm, and the exact boundary of the scab will be identified to enable further estimation of the damage ratio. The segmentation comprises the following steps: (1) Only images higher than the confidence threshold are extracted from the detection boxes for segmentation. The threshold obtained from the pre-experiment is 0.5. This value of threshold enables us to avoid a large number of pseudo image results. (2) Based on the images in the detection boxes, a new feature, i.e., RGReLU, is proposed for tea plant stress segmentation and other features, including H and RG, are generated for subsequent comparison. (3) The Otsu’s method is used to segment the scabs. (4) Lastly, the stress damage ratio of the whole image is estimated based on the results from each detection box. The flowchart of the scab segmentation method is presented in Figure 5.

It is noteworthy that the content of the images in the detection boxes is relatively diverse and may contain leaves, scabs, stems, leaf veins, and background. Moreover, the scab areas often have variable shapes, different sizes, and scattered distribution. Therefore, this work analyzes the sensitivity of scabs in different feature spaces based on color information for realizing a segmentation method with good performance, computational efficiency, and strong adaptability. The RGReLU feature is designed by computing the difference between the R channel and the G channel in the RGB color space. Then, ReLU is used to normalize the negative values to zero. The RGReLU is compared with two other features, including the features obtained after the difference between the R channel and the G channel (RG feature) and the feature of the H channel after converting the RGB image to the HIS image (H feature). In the RGB color space, the grayscale values of the three components are in the range of (0, 255). By calculating the difference between channels, the red component is strengthened whereas the green component is weakened in the RG feature. As a result, the difference between the lesion and leaf area in the image is highlighted and is conducive to the subsequent classification combined with Otsu’s method. It is worth noting that the black background in the image often becomes the transition region in the RG feature space (the eigenvalue is close to zero). Here, the RG feature space is modified by introducing the ReLU function that is commonly used in deep learning. Therefore, all the negative eigenvalues are converted to zero to avoid the possible influence of the background in the scab segmentation according to Otsu’s method. The ReLU function is mathematically expressed as follows:


(5) $$ReLU\left(x\right)=\{\begin{array}{c} x,x\geq 0\\ 0,x<0 \end{array}\;$$

In order to perform tea plant stress segmentation, simple and efficient Otsu’s method is selected. The Otsu’s method is derived from the least square method based on the histogram of a gray image, which has the best segmentation in statistical terms. Let region A and B be the two parts after threshold segmentation, $\omega_{0}$ and $\omega_{1}$ represent the probabilities of the occurrence of the A and B regions, respectively, and $\mu_{0}$ and $\mu_{1}$ represent the average gray value of regions A and B, respectively. The expression for calculating the between-class variance is as follows:


(6) $$s^{2}\left(k\right)=\omega_{0}{\left(\mu -\mu_{0}\right)}^{2}+\omega_{1}{\left(\mu -\mu_{1}\right)}^{2}\;$$

When the maximum value of $s^{2}\left(k\right)$ is obtained, the value of *k* represents the optimal threshold value.

### Transfer Learning From Canopy to UAV Detecting Scenario

The training process of deep learning models is expensive in terms of computations and requires large-scale datasets. In addition, the models usually require retraining for handling different scenarios, thus limiting the application of the models to a large extent. The transfer learning enables us to use previously learned knowledge to solve new problems (Pan and Yang, 2009). In transfer learning, only a few layers of a network are re-initialized, and the weights of other layers do not require training. The fine-tuning of network parameters makes it easy to adapt to new datasets. The transfer learning method proposed in this work consists of two stages. First, the detection model based on the TEAIMAGE dataset is obtained by fine-tuning the pre-trained model weights. Afterward, in order to investigate the effectiveness of the proposed tea plant stresses identification and segmentation strategy in terms of application, the above canopy model is transferred to the UAV image dataset to test the migration ability of the model.

### Algorithm Evaluation

The algorithm evaluation is conducted on both the object detection and the scab segmentation parts. For object detection, the mAP is used as the model evaluation index, i.e., the average value of the area under the precision-recall (PR) curve of each category, when the IoU is 0.5. This indicator comprehensively expresses the detection performance of the model, and is a good indicator of precision and recall. At the same time, the average precision (AP) of each stress is also provided for comparative analysis. The AP and mAP are mathematically expressed as:


(7) $$AP=\overset{1}{\underset{0}{\int }}P\left(R\right)d\left(R\right)\;$$

(8) $$\mathrm{mAP}=\frac{\sum_{c=1}^{C}AP\left(c\right)}{C}$$

where, *P* denotes the precision, *R* denotes the recall, and *C* denotes the number of target categories.

In the scab segmentation part, the overall accuracy (OA) is used as the evaluation index of the algorithm. The tea leaf images are visually interpreted by manually extracting the scab areas to act as a ground truth. The proposed methods are compared with the ground truth to determine the number of correctly classified and misclassified pixels for generating a confusion matrix for accuracy evaluation.


(9) $$OA=\frac{TP+TN}{TP+TN+FP+FN}$$

where, TP, FP, TN, and FN represent the true-positive, false-positive, true-negative, and false-negative pixels’ count in the segmentation result, respectively.

## Results and Discussion

### Object Detection Algorithm Result Analysis

**Figure 6:**
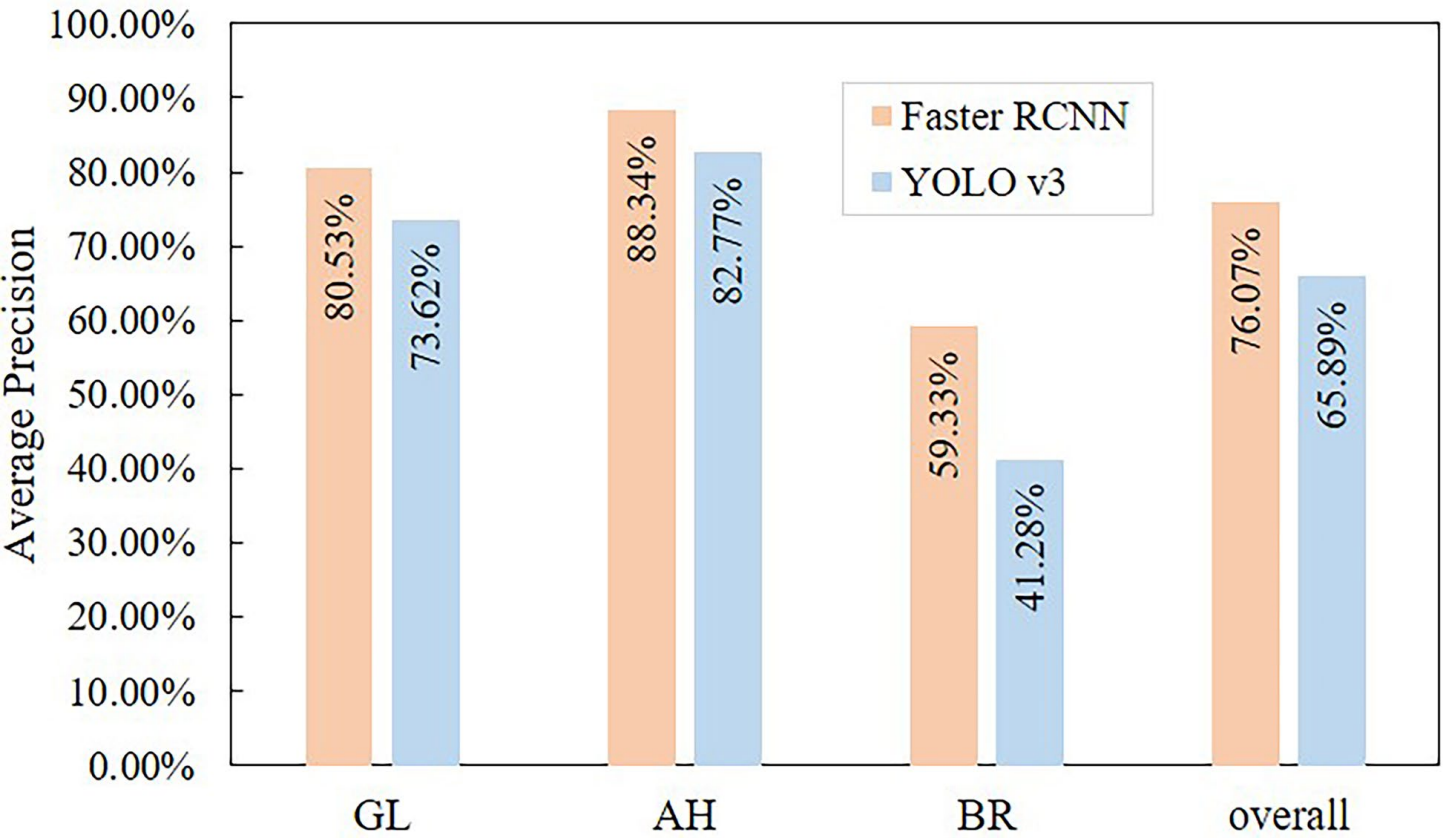
The accuracy of the object detection algorithm.

**Figure 7:**
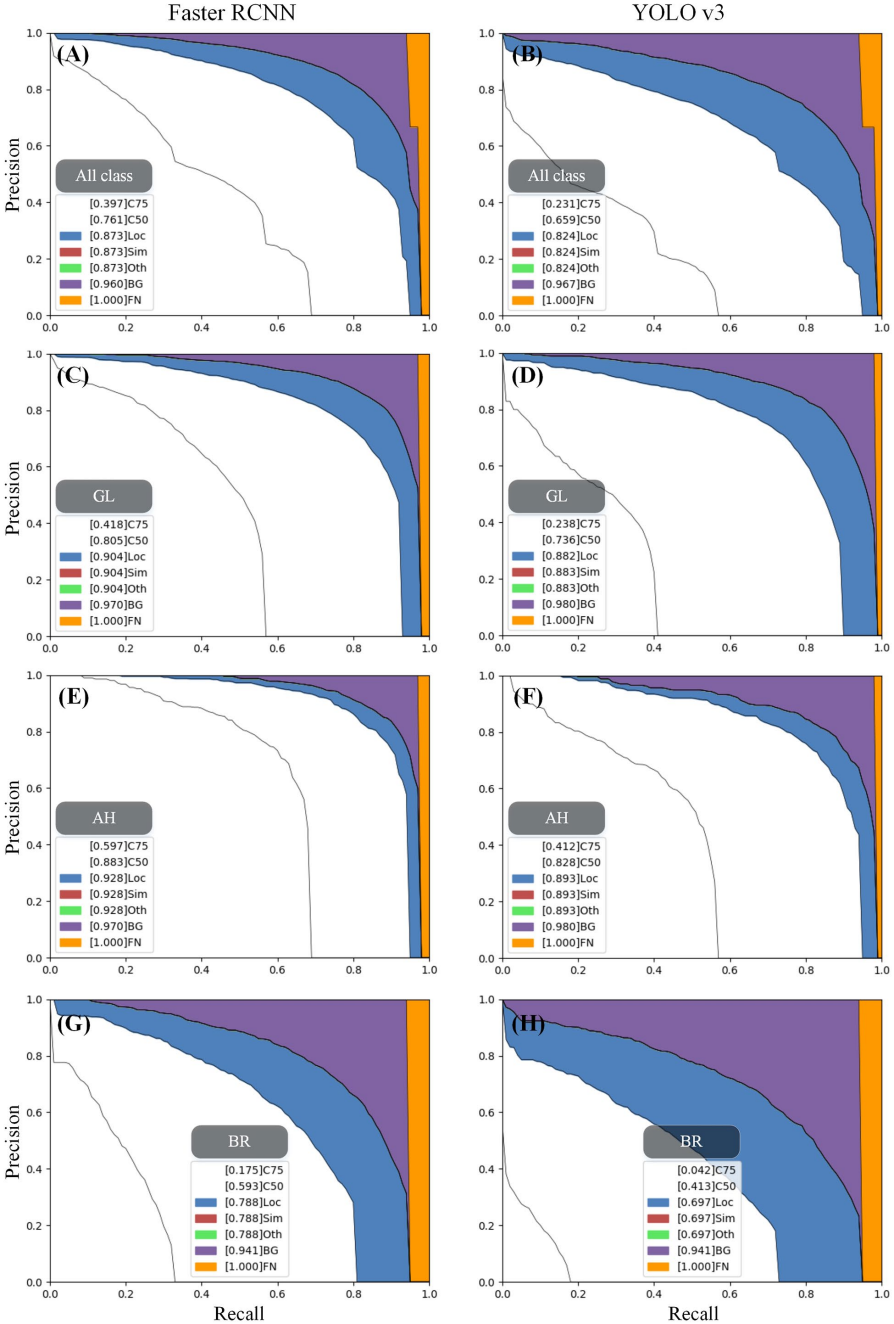
The error analysis of the object detection algorithm for each stress. The analysis of the object detection model includes seven PR curves. Due to the gradual relaxation of evaluation requirements, each curve represents a higher AP as compared to the curve presented on the left. The evaluation requirements of each PR curve are presented as: (1) C75: PR at IoU = 0.75, area under the curve corresponds to AP IoU = 0.75; (2) C50: PR at IoU = 0.50, the white area between C50 and C75 represents the AP gain due to the relaxation of IoU from 0.75 to 0.5; (3) Loc: PR at IoU = 0.10 (localization errors are ignored, but the duplicate detections are not ignored), the larger the blue area between Loc and C50, the lower is the performance of localization; (4) Sim: PR after supercategory false positives (fps) are removed. The larger the red area between Sim and Loc, the higher is the degree of confusion between super categories; (5) Oth: PR after all class confusions are removed. The larger the green area between Oth and Sim, the higher is the degree of confusion between subclasses; (6) BG: PR after all background (and class confusion) fps are removed. The larger the purple area between BG and Oth, the greater is the number of false detections in the background area; (7) FN: PR after all remaining errors are removed (trivially AP = 1). The larger the orange area between FN and BG, the more ground truth boxes are missed. The category labels of subgraphs (**A–H**) can be obtained in each gray box.

In terms of detecting the stress objects from tea canopy images, the Faster RCNN model achieves a higher accuracy (mAP = 76.07%) than YOLO v3 (mAP = 65.89%), as presented in Figure 6. The AP of Faster RCNN under three stress categories (GL: 80.53%, AH: 88.34%, and BR: 59.33%) is also higher as compared to YOLO v3 (GL: 73.62%, AH: 82.77%, BR: 41.28%). The prediction results of the two object detection algorithms are analyzed for all classes based on the methods provided by COCO.^1^ When the IoU criterion is relaxed from 0.5 to 0.1, the mAP for both object detection algorithms improves significantly (Figure 7). Among them, Faster RCNN increased by 0.112, and YOLO v3 increased by 0.165. This means that both algorithms suffer from inaccurate localization; however, the effect on YOLO v3 is higher. In Figures 7A,B, the purple area has a larger proportion as compared to the blue area. The corresponding purple area of Faster RCNN and YOLO v3 is 0.087 and 0.143, respectively, indicating that both falsely detect the background area, but YOLO v3 has higher false detection of background area. In addition, the yellow areas of Faster RCNN and YOLO v3 are small, i.e., 0.04 and 0.033, respectively, indicating that the two algorithms have low missed detections of ground truth. At the same time, the red and green areas of the two algorithms are approximately zero, indicating that there is almost no misclassification between stress categories in the two algorithms.

For three stress types, both algorithms performed best in detecting AH, then followed by GL and BR, as presented in Figure 6. The results show that the inaccurate localization and false detection of the background area significantly influence the accuracy of the model, as presented in Figures 7C–H. For instance, in Faster RCNN, the AP losses of AH, GL, and BR due to inaccurate localization are 4.5%, 9.9%, and 19.5%, respectively; the AP losses due to the false detections of background area are 4.2%, 6.6%, and 15.3%, respectively; and the AP losses due to missed detections are the lowest, i.e., 3%, 3%, and 5.9%, respectively. This may be caused by the fact that the scab areas of AH are relatively large with clear edges, which is beneficial for efficient detection. On the other hand, the scab areas in the GL image are generally small and there exist some thin-striped scabs, which pose challenges to the detection model. The presence of scabs with relatively blurry boundaries in the BR stress area makes it difficult for the model to locate scabs accurately. In addition, in BR canopy, there are some withered leaves showing similar characteristics with BR, which causes confusion for the model. Therefore, inaccurate localization and the false detection of background area have a great impact on the accuracy of YOLO v3, as presented in Figure 7.

**Figure 8:**
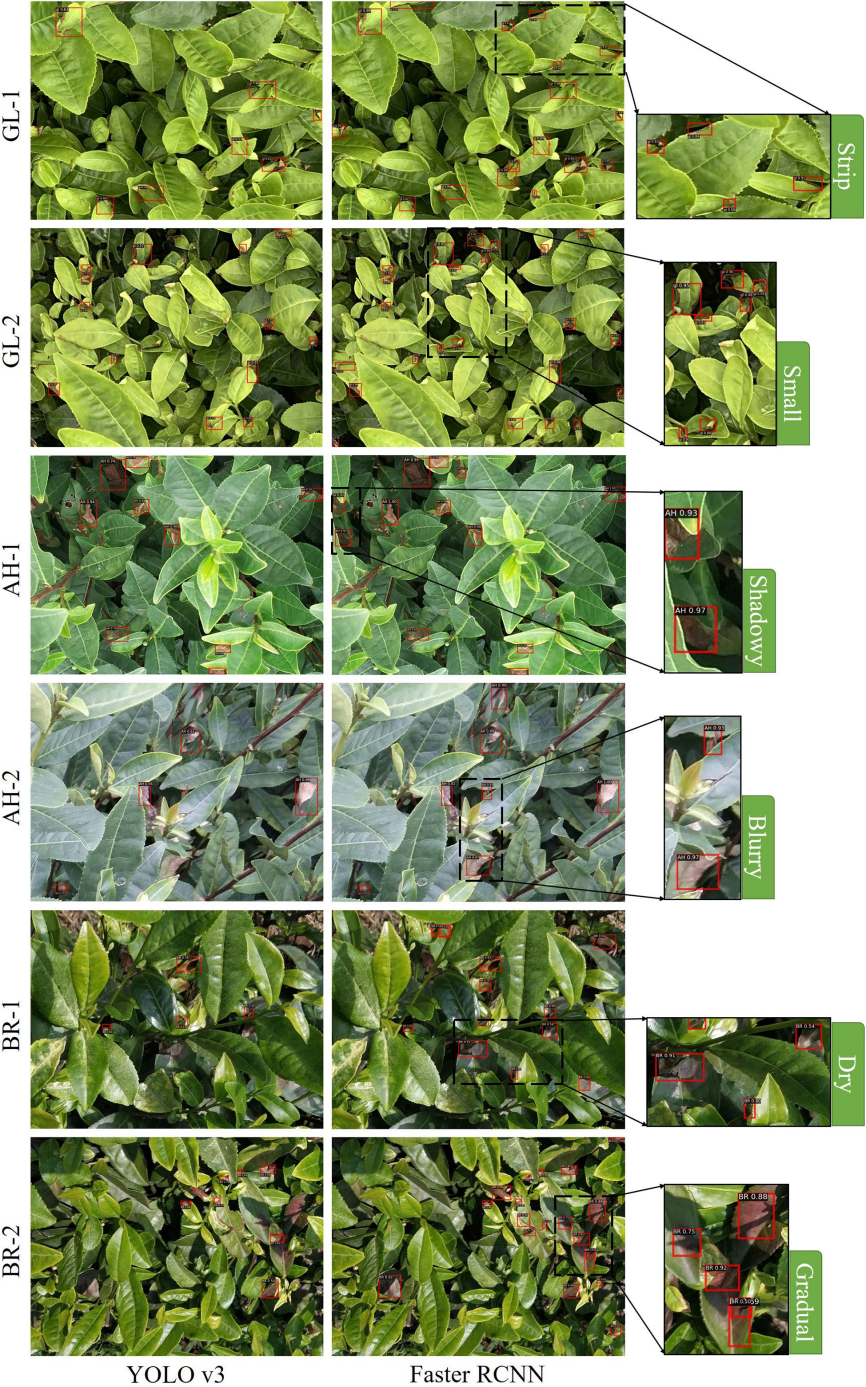
Some difficult scenarios in tea plant stress detection.

Generally, the detection accuracy of Faster RCNN is higher as compared to YOLO v3. This may be because the RPN yields more balanced positive and negative samples in the model. As a two-staged object detection algorithm, Faster RCNN splits the foreground and background in the RPN and performs preliminary target localization. After obtaining the foreground and background regions, the IoU-balanced sampling method is used to screen out the more balanced positive and negative samples (Pang et al., 2019). Then, the obtained proposal boxes are classified and more accurate border regression is performed. On the contrary, as a single-stage algorithm, YOLO v3 tends to generate too many negative samples and very few positive samples, which makes it difficult for the network to learn effective information. The detection results of the two algorithms at the canopy scale are presented in Figure 8. Among them, Faster RCNN performs efficiently and completes the identification and localization of different stress lesions, while YOLO v3 does not perform well under such a complex scenario. For instance, in case of very small scabs (GL-1), irregular thin strips (GL-2), and scabs with blurry edges (BR-2), the detection performance of Faster RCNN is better as compared to YOLO v3. This may be an effect caused by the use of deformable convolution kernels in the backbone network of Faster RCNN. The deformable convolution allows the sampling points to diffuse into irregular shapes, which better adapts the complex image geometric transformations, and shows advantages for targets with irregular sizes and shapes of tea plant stress. In addition, this work uses IoU-balanced sampling algorithm in RPN, which enables the Faster RCNN to learn more hard samples. When the scabs are in abnormal states, such as shadow (AH-1), blur (AH-2), and dry (BR-1), the model still achieves ideal detection results. While the mAP of HRNet-based Faster RCNN without IoU balance sampling and deformable convolution is 74.64%. Therefore, the specified Faster RCNN is used as the object detection algorithm in this work. It is worth noting that although the accuracy of the specified Faster RCNN is not very high (mAP = 76.07%), most tea plant stresses are successfully detected. The deviation of detected boxes may account for a certain error rate. However, the goal in this step is to identify where the stresses occur in the canopy, instead of obtaining the precise locations of the detection boxes. Therefore, such deviation does not affect the practical application of the detection results. Moreover, more precise scab regions are generated in the subsequent image analysis step, which only requires a rough location of the detected boxes.

### Stress Segmentation of Tea Plant Based on Image Analysis

**Figure 9:**
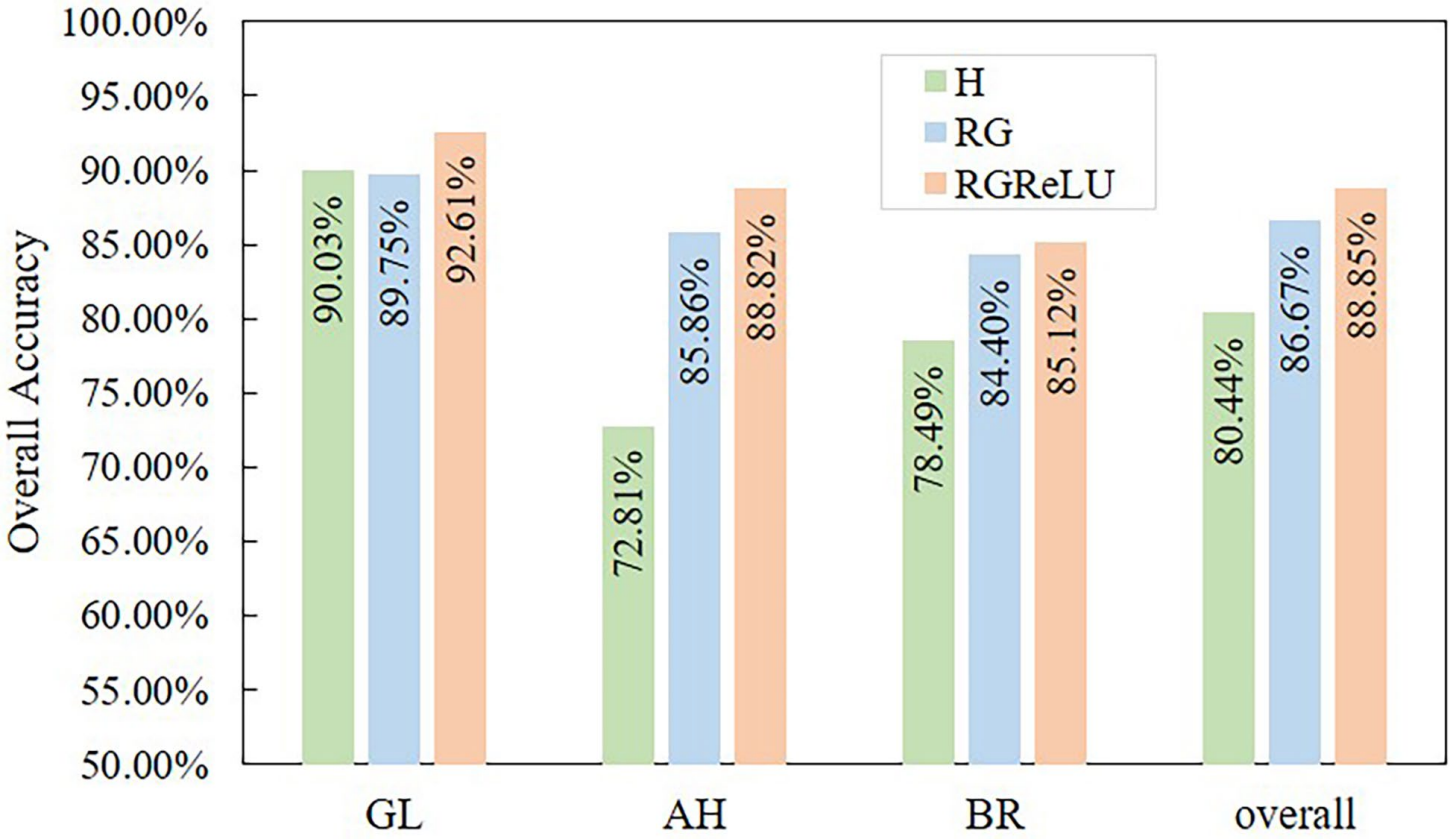
The overall accuracy of scab segmentation.

**Figure 10:**
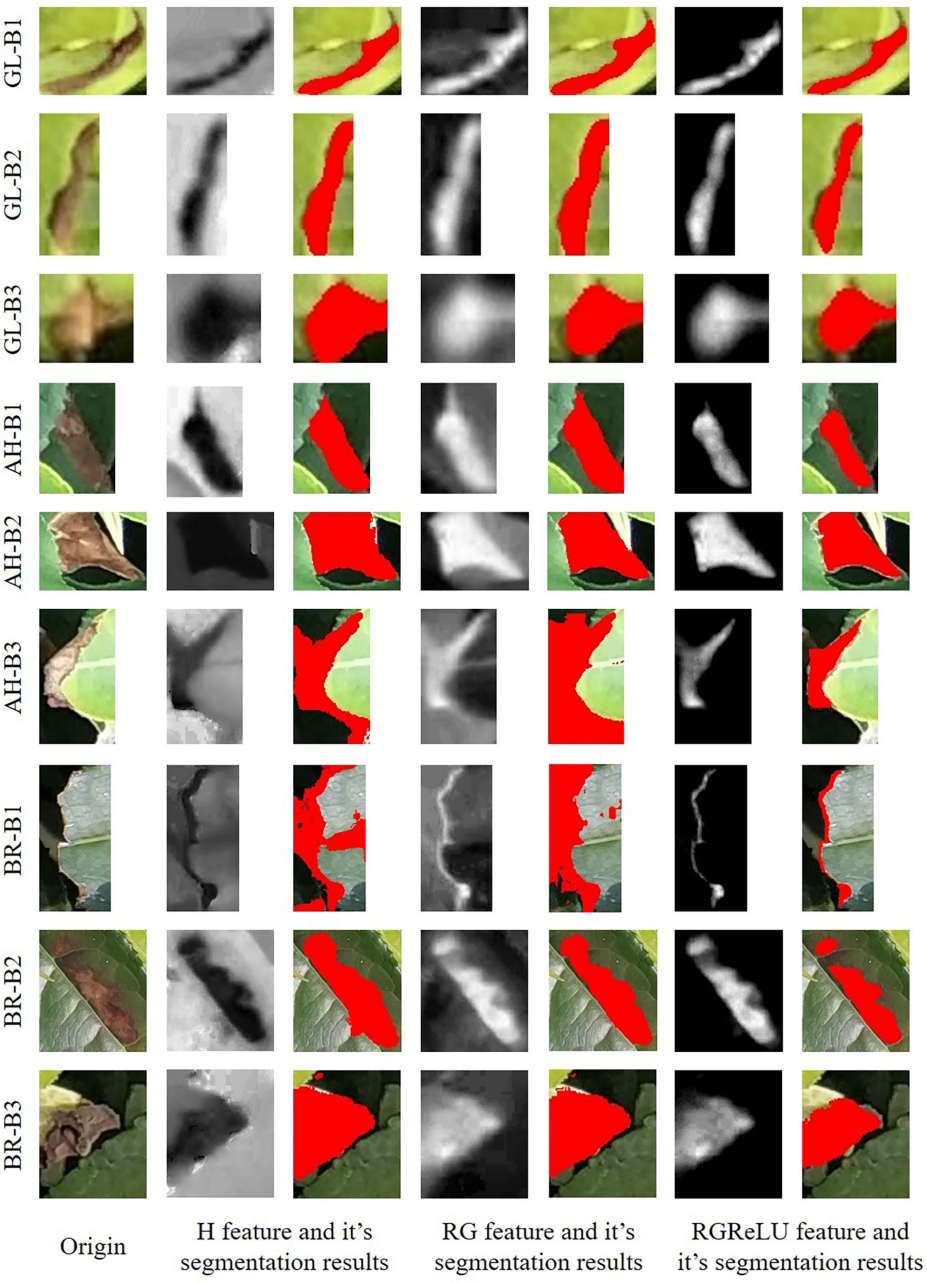
The segmentation results of tea plant stress by using different features. GL-B, AH-B, and BR-B denote the detection box images of tea green leafhopper, anthracnose, and sunburn, respectively.

The images extracted from the detection boxes are analyzed to obtain accurate scab segments for calculating the damage ratio of each stress type. The different features used in this study show different performance in scab segmentation, as presented in Figure 9. The algorithm based on RGReLU features has the highest accuracy (OA = 88.85%), followed by RG (OA = 86.67%), and H features (OA = 80.44%). Please note that the features based on RGB color space show high sensitivity to stress, as they capture the visual traits of scabs under real environment. The analysis of image information in the detection box shows that the boxes mainly include stress scabs (basically red, brown, and pink), normal leaves (green), and background areas (black). Therefore, by taking the difference between the R and G channels, the red channel feature in the image is strengthened (the red corresponds to the RG feature value of 255), while the green channel feature is weakened (the green corresponds to the RG feature value of −255), and the black channel feature becomes the middle zone in the RG color feature (the black corresponds to the RG feature value of 0) for effectively separating the scabs, leaves, and the background. However, the actual situation is often more complicated, and, sometimes, there are no ideal red, green, and black areas, as presented in Figure 10. For example, when the color of the scab is white due to illumination, reflection, etc., the red channel feature is weakened and the RG eigenvalue is reduced, thus making it difficult for the Otsu’s method to effectively distinguish the scab from the background (such as AH-B3 and BR-B1). It is worth noting that even for canopy images with complicated circumstances, the RGReLU feature proposed in this work still achieves satisfactory performance in segmenting the stressed regions. This may be because the negative part of the RG eigenvalue is uniformly changed to zero by using the ReLU function, so the eigenvalues of the regions where the red channel feature is weaker than the green channel feature (leaf areas, leaf veins, etc.) are all zeroed, thus integrating into the background region, which is beneficial for the subsequent extraction of the damaged area by using the Otsu’s method (2.18% higher than the RG feature accuracy). In addition, Figure 10 shows that the RGReLU feature has better segmentation performance for three types of stress scabs as compared to the other two features under different detection backgrounds, which delineates the scab edges effectively. Contrary, the H and RG features yield large segmented scab areas, such as GL-B1, GL-B2, GL-B3, AH-B1, and BR-B2.

The segmentation effect of each stress type is analyzed. The results show that the three features have the best segmentation ability for GL (OA over 90%), followed by AH, and BR. This is because the GL contains a single color that is closer to red and has high sensitivity in RG features. There are some brown and bright yellow scabs in the AH samples, which degrade the detection results. For BR stress, the color of the scab area is moderate, without clear edges, and the lesion is prone to dry and blackening, resulting in the loss of some accuracy.

**Figure 11:**
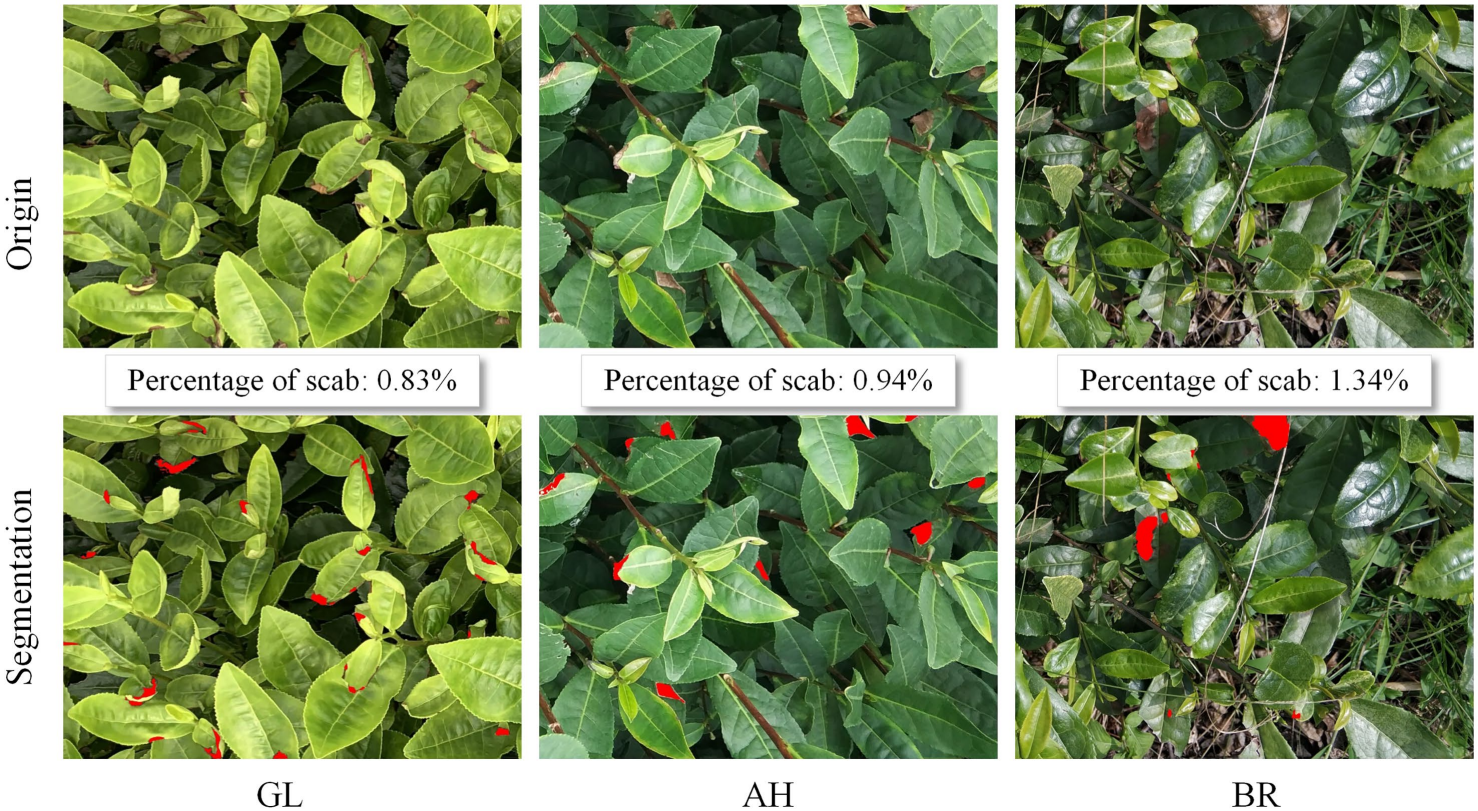
The illustration of tea plant stress segmentation at canopy scale.

The deep learning and image processing stepwise recognition strategy proposed in this work at the canopy scale overcomes the difficulties faced by a single image processing technique in dealing with various complex local changes in the canopy, such as illumination, shadow, blur, occlusion, etc. In addition to automatic segmentation of the scab areas, the proposed method also provides the proportion of stress infection, which is an important indicator for prevention practice. The proportion of scabs for AH, BR, and GL samples are 0.83%, 0.94%, and 1.34%, respectively, as presented in Figure 11. On the other hand, as compared to the mask detection deep learning approach, the proposed method only requires the labeled boxes for training, instead of providing the exact object boundaries. In this way, the detailed edge information of stressed regions is obtained in a computationally efficient manner, which facilitates the derivation of canopy level scab proportion.

### Transfer Learning From Canopy to UAV Detecting Scenarios

**Figure 12:**
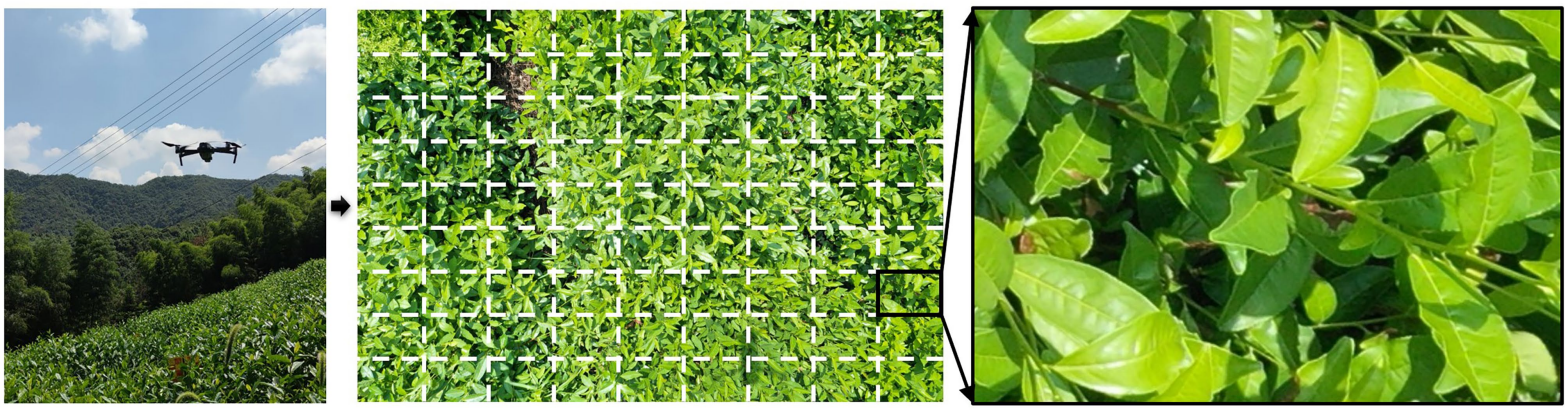
The demonstration of unmanned aerial vehicle (UAV) image and image fragmentation of tea plant stress.

**Figure 13:**
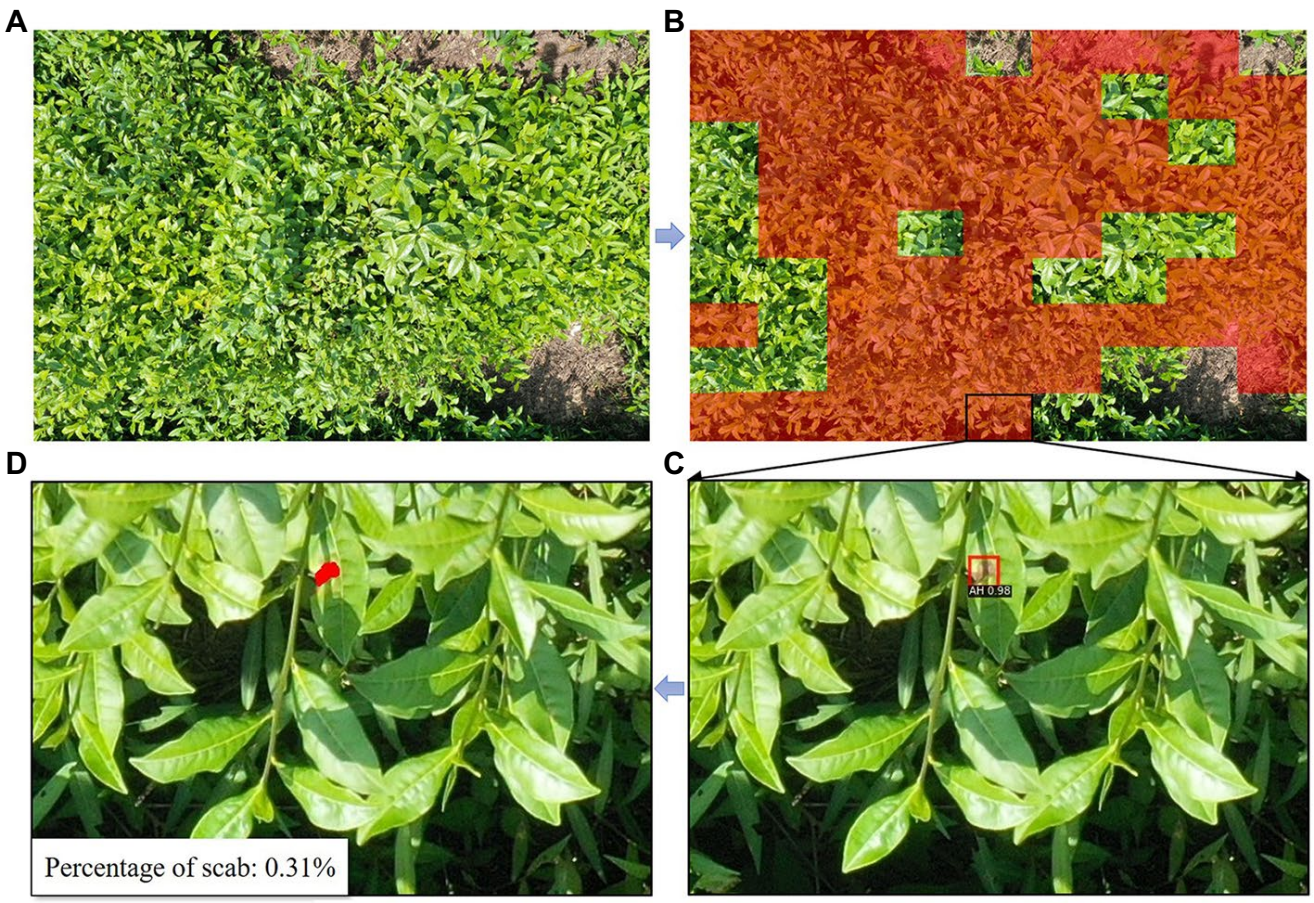
Precise scab detection and labeling in UAV images by transfer learning. **(A)** The original UAV image; **(B)** stressed sub-areas highlighted in red; **(C)** sub-image with stressed area labeled in a box; and **(D)** sub-image with an exact boundary of stressed area.

In this work, in order to evaluate the application potential of the proposed method in large-scale scenarios, the UAV (DJI Mavic) is used to collect the RGB images of AH, the UAV height is 3 m, and the UAV camera is Hasselblad (L1D-20c) with a resolution of 5,472 × 3,648. After cropping the original image, 100 sub-images containing scabs are obtained for fine-tuning the model, as presented in Figure 12. The model is fine-tuned for the UAV image dataset based on the Faster RCNN network parameters. The results show that the model converges quickly and achieves an appropriate accuracy (mAP = 86.48%). The segmentation results of the resulting model for the UAV images are presented in Figure 13. The entire image is uniformly fragmented and the resulting sub-images are analyzed. The transparent red regions show that the corresponding image pieces are infected by stresses. Then, the scabs in each infected image piece are segmented based on the aforementioned image processing technique. The resulting scab regions are used for computing the damage ratio of the entire UAV image. Please note that the transferred model achieves rapid detection of stresses, differentiation of stress types, and scab recognition, which indicates a strong generalization ability of the proposed method. The model application of such a strategy using the UAV images enables the automatic scouting of stresses in a wide area of tea garden. With the fast development of UAV systems, the automatic UAV techniques are getting more and more mature and cost-effective. This promotes the application of the tea plant stress detection method in multiple scenarios, such as early warning and control of diseases and pests, plants phenotyping for breeding, etc. Moreover, similar strategies and approaches can also be introduced in stress detection in orchards and other economic crops planted in open areas.

## Conclusion

Aiming at automatic scab segmentation and damage ratio assessment of tea plant canopy images for tea stress detection, this work proposes an intelligent segmentation strategy that synergizes deep learning and image processing. The proposed method achieves automatic recognition, differentiation of different types of stresses, and obtains the precise boundaries of all stress scabs for deriving the accurate damage ratio. The specified Faster RCNN presented in this work uses deformable convolution kernels and IoU-balanced sampling to effectively detect the three typical tea plant stresses of tea green leafhopper, anthracnose, and sunburn. And the performance of the specified Faster RCNN (mAP = 76.07%) is better as compared to YOLO v3 (mAP = 65.89%) under complicated scenarios (illumination, shadow, blur, occlusion, etc.) In order to extract the boundaries of tea plant scabs in the detection box, the RGReLU feature is used in an image processing procedure, which enhances the difference between the background and the stressed area. This stepwise strategy effectively reduces the complexity of tea plant stress segmentation in practical scenarios. And the generated canopy-scale model can be transferred to the UAV images, which shows the potential to apply the proposed model for scouting stresses in large-area tea gardens.

## Data Availability Statement

The raw data supporting the conclusions of this article will be made available by the authors, without undue reservation.

## Author Contributions

XZ, JZ, and DC conceived the idea, proposed the method, and revised the paper. JZ and LYu contributed to the preparation of equipment and acquisition of data. XZ and AT wrote the code and tested the method. AT, YY, and LYa validated results. XZ wrote the paper. All authors contributed to the article and approved the submitted version.

## Funding

This work was supported by the National Key R&D Program of China (2019YFE0125300), the National Natural Science Foundation of China (grant no. 42071420), and the Major Special Project for 2025 Scientific and Technological Innovation (Major Scientific and Technological Task Project in Ningbo City; 2021Z048).

## Conflict of Interest

The authors declare that the research was conducted in the absence of any commercial or financial relationships that could be construed as a potential conflict of interest.

## Publisher’s Note

All claims expressed in this article are solely those of the authors and do not necessarily represent those of their affiliated organizations, or those of the publisher, the editors and the reviewers. Any product that may be evaluated in this article, or claim that may be made by its manufacturer, is not guaranteed or endorsed by the publisher.

## Footnotes

^1^ https://cocodataset.org/#detection-eval

## Abbreviations

AH: Anthracnose
GL: Tea green leafhopper
BR: Leaf sunburn
Faster RCNN: Faster region-based convolutional neural network
YOLO: You only look once
UAV: Unmanned aerial vehicle
mAP: Mean average precision
OA: Overall accuracy
SVM: Support vector machine
RF: Random forest
CNN: Convolutional neural network
RCNN: Region-based convolutional neural network
RPN: Regional proposal network
IoU: Intersection over union
ReLU: Rectified linear unit
PR: Precision-recall
AP: Average precision
TP: True positive
FP: False positive
TN: True negative
FN: False negative
